# Does size matter? Comparison of miniaturized (22 Fr) versus standard (26 Fr) instruments in pulsed thulium:YAG laser enucleation of the prostate

**DOI:** 10.1007/s00345-026-06204-8

**Published:** 2026-01-11

**Authors:** Ahmet Furkan Ozsoy, Mehmet Erol Maras, Yusuf Huzeyfe Sahin, Atakan Atakli, Emre Erdem, Atakhan Musayev, Muhammed Arif Ibis, Cagri Akpinar, Mehmet Ilker Gokce

**Affiliations:** Department of Urology, School of Medicine, Ankara University, Ankara, Turkey

**Keywords:** MiLEP, AEEP, ThuLEP, Urethral stricture, Postoperative incontinence

## Abstract

**Purpose:**

Anatomic enucleation of the prostate is increasingly performed worldwide. While 26-Fr instruments are commonly used, miniaturized devices have been associated with fewer complications and improved recovery without compromising efficiency. We compared intraoperative and postoperative outcomes between 22-Fr and 26-Fr instruments in pulsed Thulium:YAG anatomic enucleation.

**Methods:**

A total of 150 patients who underwent pulsed Thulium:YAG laser enucleation of the prostate between December 2024 and July 2025 were prospectively enrolled. MiLEP was performed in 70 patients, while standard AEEP was performed in 80. The primary endpoint was intraoperative efficiency, and secondary endpoints were postoperative complications and functional recovery, comparing miniaturized with standard instruments.

**Results:**

The MiLEP group had a smaller median prostate volume (50.5 vs. 88 mL, *p* < 0.001) and shorter enucleation time (19 vs. 25 min, *p* < 0.001), although enucleation efficiency was higher in the standard group (3.6 vs. 2.7 g/min, *p* < 0.001). Irrigation volume was lower (13.5 vs. 21 L, *p* < 0.001), and no intraoperative urethrotomy/meatotomy was required in the MiLEP group compared with 10 patients (12.5%) in the standard group (*p* = 0.002). At 1 month, urethral stricture was identified in 1 (1.4%) MiLEP and 3 (3.8%) standard patients (*p* = 0.379), whereas incontinence occurred in 3 (4.3%) and 4 (5.0%), respectively (*p* = 0.836).

**Conclusions:**

MiLEP with pulsed Thulium:YAG laser represents an effective modality for anatomic enucleation of the prostate. It was associated with comparable intraoperative efficiency and favorable postoperative functional outcomes compared to standard resectoscope size. Our findings suggest that MiLEP may be a suitable option for selected patients, particularly those with lower urethral compliance and smaller prostate volume.

## Introduction

Transurethral resection of the prostate (TURP) has long been regarded as the benchmark for the surgical management of small- and medium-sized prostates [1]. However, since its first description by Gilling in 1998, anatomic laser enucleation of the prostate (AEEP) has gradually gained popularity and widespread acceptance among urologists [2].

Moreover, for large prostates, AEEP provides efficacy comparable to open prostatectomy while offering shorter catheterization time, reduced hospital stay, and decreased blood loss, thereby establishing itself as a minimally invasive yet effective alternative [3]. Consequently, International guidelines such as the EAU, AUA, and NICE have endorsed laser AEEP as a size-independent surgical approach [4]. Although laser enucleation of the prostate is associated with low morbidity rates, the occurrence of transient urinary incontinence (up to 13%) [5] and urethral stricture (up to 1.7%) [6] may decrease overall satisfaction for both surgeons and patients. Several factors have been suggested to influence these complication rates, and instrument size has been considered an independent factor [7, 8].

With the idea of adapting the instruments to the urethra and not vice versa, Figueiredo developed miniaturized equipment for AEEP [9]. This procedure, termed ‘miniaturized laser enucleation of the prostate’ (MiLEP), has been evaluated in only a limited number of clinical studies and in none of these studies the primary energy source was the new generation pulsed Thulium:YAG. Therefore, in this study we aimed to compare the outcomes of MiLEP and standard sized AEEP, operated with the pulsed Thulium:YAG laser prospectively.

## Patient and methods

### Study design

A total of 150 patients who underwent pulsed Thulium:YAG laser enucleation of the prostate between December 2024 and July 2025 were prospectively enrolled. All procedures were performed by a single surgeon who had experience with more than 2500 AEEP cases. The choice of surgical technique was determined preoperatively by the surgeon, and patients were subsequently allocated to groups in a non-randomized manner based on the availability of surgical equipment (e.g., sterilization-related issues) prior to surgery.

**Fig. 1:**
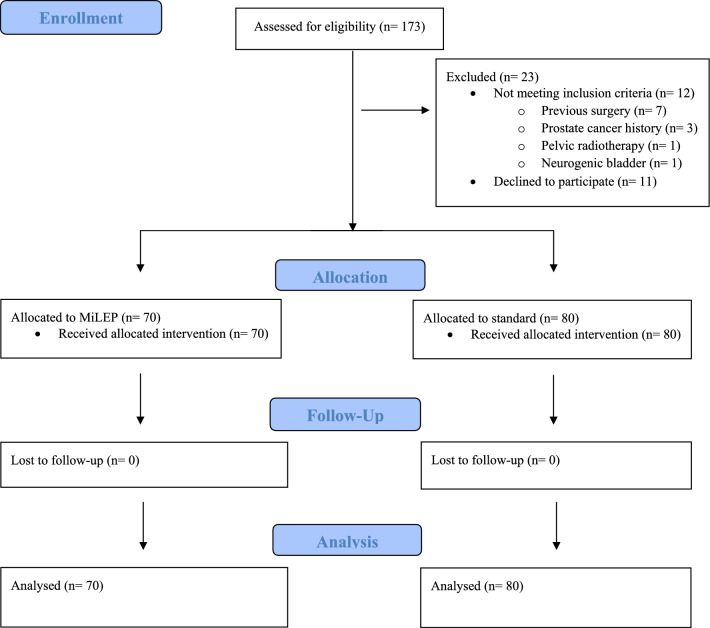
Flow diagram of patient enrollment, exclusion, and allocation

Patients with lower urinary tract symptoms (LUTS) who had inadequate response or worsening symptoms after medical therapy, acute urinary retention (AUR), or any other absolute indication for surgery (recurrent urinary tract infections, bilateral hydronephrosis with renal impairment, or recurrent hematuria due to BPH) were eligible for inclusion. Patients with a history of previous prostate or urethral surgery, prostate cancer, pelvic radiotherapy, or neurogenic bladder were excluded (Fig. 1). Demographic characteristics; preoperative patient-based factors such as prostate-specific antigen (PSA), prostate volume assessed by ultrasonography (US) or magnetic resonance imaging (MRI), perioperative variables, International Prostate Symptom Score (IPSS), and uroflowmetry parameters; as well as intraoperative and postoperative follow-up parameters were collected.

Enucleation time was defined as the interval between the initiation of enucleation and the start of morcellation, whereas surgical time was defined as the period from cystoscopy to catheter placement.

Enucleation efficiency (g/min) was calculated by normalizing prostate volume to enucleation time. Complications were recorded according to the Clavien Dindo classification system [10].

All procedures were performed in accordance with the 1964 Helsinki declaration and its later amendments. Institutional ethics committee approval was obtained (Application No: 2024000746-2; Decision No: i01-09-25).

### Surgical procedure

In the standard group, enucleation was performed with a 26 Ch resectoscope set (RZ Medizintechnik, Tuttlingen, Germany or Karl Storz, Tuttlingen, Germany). In MiLEP group, a 22 Ch resectoscope set (RZ Medizintechnik, Tuttlingen, Germany) was used. Morcellation was performed with Multicut morcellator (Asclepion Laser Technologies GmbH, Jena, Germany). For both groups, a 100-W pulsed Thulium:YAG laser (Dornier Thulio®, Dornier MedTech Systems GmbH, Wessling, Germany) with a 550-μm end-firing laser fiber was employed. When MiLEP was performed for enucleation, laser settings were 1.5 J at 50 Hz (75 W). In procedures conducted with 26 Fr equipment, settings were 2 J at 50 Hz (100 W). In both groups for coagulation the laser settings were 0.6 J and 50 Hz. Laser settings were standardized and were not adjusted intraoperatively.

The surgeries were performed either with the en-bloc or two lobes techniques and the choice of enucleation technique was decided intraoperatively according to prostate volume and anatomy. Following surgery, a 20 Fr Foley catheter was inserted, and continuous bladder irrigation was commenced. The catheter was routinely removed on the second postoperative day.

### Postoperative follow-up

At the first postoperative month, all patients underwent evaluation, including uroflowmetry and International Prostate Symptom Score (IPSS) assessment. Postoperative stress incontinence (SI), defined as any patient-reported urine loss on a pad, was also recorded. At the sixth postoperative month, all patients were evaluated either in the outpatient clinic or via telephone through symptom-based assessment. At the sixth month follow-up, only patients who reported lower urinary tract symptoms underwent uroflowmetry.

### Statistical analysis

All statistical analyses were performed using R version 4.1.2 (R Foundation for Statistical Computing, Vienna, Austria), with *p* < 0.05 considered statistically significant. The Shapiro–Wilk test was used to assess normality. Continuous variables were reported as median and interquartile range (IQR) or mean and standard deviation, while categorical variables were reported as absolute frequencies and percentages. Patient demographics, perioperative parameters, and postoperative outcomes were compared between the MiLEP and standard groups using the Chi-square test or Fisher’s exact test for categorical variables, and the Mann–Whitney U test or independent t-test for continuous variables. Multivariable linear regression analyses were performed using four separate models to evaluate intraoperative parameters that showed meaningful differences between the two groups. Covariates were selected based on variables that differed significantly between the groups and were clinically relevant in routine practice. Due to high collinearity between prostate volume and enucleation efficiency, prostate volume was not included in the enucleation efficiency model.

## Results

MiLEP was performed in 70 patients, while standard AEEP was performed in 80 patients. The mean age was 65.7 ± 9.2 years in the MiLEP group and 67.7 ± 7.4 years in the standard group (*p* = 0.147). Demographic characteristics were comparable between the two groups (Table 1). The median prostate volume was 50.5 (34.5–77) g in the MiLEP group and 88 (86.7–137.7) g in the standard group (*p* < 0.001). The median preoperative total PSA level was also lower in the MiLEP group compared to the standard group (2.3 vs. 4.4 ng/mL, *p* < 0.001). Enucleation type was comparable between the two groups (Table 2). The median total energy was 83.6 (64.7–102.2) kJ in the MiLEP group and 111.2 (70.4–181.1) kJ in the standard group (*p* = 0.005). The median enucleation time was shorter in the MiLEP group compared with the standard group (19 vs. 25 min, *p* < 0.001); however, the enucleation efficiency (g/min) was higher in the standard group (2.7 vs. 3.6, *p* < 0.001).

**Table 1:** Demographic characteristics and preoperative clinical assessments

	MiLEP (22 Fr) *n* = 70	Standard (26 Fr) *n* = 80	*p* value
Age (years), mean ± SD	65.7 ± 9.2	67.7 ± 7.4	0.147
BMI			0.457
18.5–24.9	21 (30%)	24 (30%)	
25–29.9	37 (52.9%)	39 (48.8%)	
30–34.9	12 (17.1%)	13 (16.3%)	
35–39.9	0	3 (3.8%)	
> 40	0	1 (1.3%)	
Hypertension, n (%)	29 (41.4%)	40 (50%)	0.293
ASA-score			0.137
ASA 1	22 (31.4%)	14 (17.5%)	
ASA 2	41 (58.6%)	56 (70%)	
ASA 3	7 (10%)	10 (12.5%)	
Alpha blocker, n (%)	54 (77.1%)	60 (75%)	0.759
5- alpha reductase inhibitor, n (%)	2 (2.9%)	9 (11.3%)	0.049^*^
Preoperative incontinence	18 (25.7%)	16 (20%)	0.404
Prostate Volume (g), median (IQR)	50.5 (34.5–77)	88 (86.7–137.7)	< 0.001^**^
PSA (ng/mL), median (IQR)	2.3 (1.1–4.2)	4.4 (2.7–6.9)	< 0.001^**^
Preoperative IPSS			0.557
Mild	4 (5.7%)	8 (10%)	
Moderate	33 (47.1%)	33 (41.3%)	
Severe	33 (47.1%)	39 (48.8%)	
Preoperative catheter ındwelling	14 (20%)	18 (22.5%)	0.709
Preoperative Qmax (mL), median (IQR)	11.5 (7.6–15.5)	9 (6–11.7)	0.057
Preoperative PVR (mL), median (IQR)	145.5 (60–321.5)	111 (48.5–322.5)	0.885

*MiLEP* Miniaturized laser enucleation of prostate, *SD* Standard deviation, *BMI* Body mass index, *ASA* American society of anesthesiologists, *IQR* Interquartile range, *PSA* Prostate spesific antigen, *ng* nanogram, *mL* milliliter *IPSS* International prostate symptom score, *Qmax* Maximum urinary flow rate, *PVR* Postvoidal residual

^*^Chi-square test

^**^Mann–Whitney U test

**Table 2:** Comparison of perioperative and postoperative features

		MiLEP (22 Fr) n = 70	Standard (26 Fr) n = 80	*p* value
Total energy (kJ), median (IQR)		83.6 (64.7–102.2)	111.2 (70.4–181.1)	0.005^*^
Enucleation time (min), median (IQR)		19 (15–21.5)	25 (18.5–33.7)	< 0.001^*^
Morcellation time (min), median (IQR)		10 (5–18.2)	20 (10.2–33.7)	< 0.001^*^
Operation time (min), median (IQR)		35 (28.7–49)	55 (40–82.2)	< 0.001^*^
Enucleation efficiency (g/min), median (IQR)		2.7 (2.2–4.2)	3.6 (2.7–5.9)	< 0.001^*^
Enucleation type				0.099
Enbloc		26 (37.1%)	17 (21.3%)	
Bilober		41 (58.6%)	59 (73.8%)	
Trilober		3 (4.3%)	4 (5%)	
Early apical release, n (%)		48 (68.6%)	63 (78.8%)	0.156
Irrigation consumption (L), median (IQR)		13.5 (9.5–18)	21 (18–30)	< 0.001^*^
Need for meatotomy/urethrotomy intraoperatively, n (%)		0	10 (12.5%)	0.002^*^
Postoperative Qmax (mL), median (IQR)		24 (23–26.2)	25.3 (23–27)	0.2
Postoperative IPSS (1.month)				0.31
Mild		61 (87.1%)	66 (82.5%)	
Moderate		9 (12.9%)	14 (17.5%)	
Severe		0	0	
Clavien–Dindo grade complications				
*Grade 2*				
Fever		1 (1.4%)	1 (1.3%)	0.924
Recatheterization		2 (2.9%)	2 (2.6%)	0.901
Prolongod irrigation		1 (1.4%)	2 (2.5%)	0.64
*Grade 3b*				
Gross hematuria (intervention required)		0	1 (1.3%)	0.348
Postoperative acute urinary retention, n (%)		2 (2.9%)	2 (2.6%)	0.901
Urethral stricture (1 month), n (%)		1 (1.4%)	3 (3.8%)	0.379
Postoperative incontinence (1 month), n (%)		3 (4.3%)	4 (5%)	0.836
Incontinence type				0.19
Urge		1 (1.4%)	2 (2.5%)	
Stress		1 (1.4%)	2 (2.5%)	
Mix		1 (1.4%)	0	
Urethral stricture (6 month), n (%)		2 (2.9%)	4 (5%)	0.504
Postoperative incontinence (6 month), n (%)		0	1 (1.3%)	0.348
Incontinence type (6 month), n (%)				1.0
Urge		0	1 (1.3%)	
Stress		0	0	
Mix		0	0	

*MiLEP* Miniaturized laser enuclation of prostate, *kJ* kilojoule, *IQR* Interquartile range, *g* gram, *min* minutes, *L* Liter (s), *mL* milliliter, *IPSS* International prostate symptom score

^*^Mann–Whitney U test

In addition, both the median morcellation time (10 vs. 20 min, *p* < 0.001) and the median operation time (35 vs. 55 min, *p* < 0.001) were significantly shorter in the MiLEP group than in the standard group. The median irrigation volume was lower in the MiLEP group than in the standard group (13.5 vs. 21 L, *p* < 0.001). While no intraoperative urethrotomy or meatotomy was required in the MiLEP group, urethrotomy or meatotomy was performed in 10 patients (12.5%) in the standard group (*p* = 0.002), as the 22-Fr miniaturized equipment was able to traverse pre-existing urethral narrowing without the need for additional intervention.

The perioperative complication rates were also comparable between the two groups (Table 2). Hematuria requiring transfusion or readmission to the hospital was not required in any patients in both groups. At 1 month postoperatively, urethral stricture was observed in 1 patient (1.4%) in the MiLEP group and in 3 patients (3.8%) in the standard group (*p* = 0.379). Postoperative urinary incontinence occurred in 3 patients (4.3%) in the MiLEP group and in 4 patients (5.0%) in the standard group (*p* = 0.836).

At 6 months postoperatively, urethral stricture was observed in 2 patients (2.9%) in the MiLEP group and in 4 patients (5.0%) in the standard group (*p* = 0.504). At the 6-month follow-up, no patients in the MiLEP group reported urinary incontinence, whereas urge urinary incontinence was observed in 1 patient (1.3%) in the standard group (*p* = 0.348).

In Model 1, multivariable linear regression analysis demonstrated that resectoscope size (26 vs 22) was independently associated with longer operating time (β = 0.16, 95% CI 1.65–18.49, *p* = 0.019), while prostate volume showed a stronger independent association with operating time (β = 0.54, 95% CI 0.16–0.27, *p* < 0.001). In Model 2, resectoscope size was independently associated with higher enucleation efficiency (β = 0.20, 95% CI 0.07–0.73, *p* = 0.019). In Model 3, both operation time (β = 0.28, 95% CI 0.03–0.14, *p* = 0.002) and resectoscope size (β = 0.23, 95% CI 1.36–7.22, *p* = 0.004) were independently associated with higher irrigation fluid consumption. Finally, in Model 4, prostate volume (β = 0.21, 95% CI 0.28–0.84, *p* = 0.004) and enucleation time (β = 0.51, 95% CI 1.44–2.52, *p* < 0.001) were independently associated with higher total energy consumption (Table 3).

**Table 3:** Multivariable linear regression analyses of intraoperative parameters

Parameters	B	SE	t	p	95% CI	β
*Model 1: Operating time*						
Resectoscope size (26 vs 22)	10.07	4.26	2.36	**0.019**	[1.65, 18.49]	0.16
Prostate volume	0.22	0.03	7.70	** < 0.001**	[0.16, 0.27]	0.54
Age	−0.19	0.25	-0.77	0.44	[−0.68, 0.3]	−0.05
Preoperative catheter indwelling time	6.98	4.98	1.40	0.163	[−2.85, 16.82]	0.09
Enucleation type						
Enbloc	Ref					
Bilober	5.10	4.52	1.13	0.261	[−3.84, 14.04]	0.08
Trilober	17.29	9.85	1.76	0.081	[−2.18, 36.76]	0.12
*Model 2: Enucleation Efficiency*						
Resectoscope size (26 vs 22)	0.402	0.169	2.38	**0.019**	[0.07, 0.73]	0.20
Age	0.012	0.010	1.19	0.235	[−0.008, 0.03]	0.10
Enucleation type						
Enbloc	Ref					
Bilober	0.125	0.188	0.66	0.507	[−0.24, 0.49]	0.06
Trilober	−0.505	0.415	−1.22	0.225	[−1.32, 0.31]	−0.10
Preoperative catheter indwelling time	0.119	0.209	0.57	0.569	[−0.29, 0.53]	0.05
*Model 3: Irrigation consumption*						
Prostate volume	0.015	0.011	1.37	0.173	[−0.007, 0.03]	0.13
Operation time	0.087	0.028	3.08	**0.002**	[0.03, 0.14]	0.28
Resectoscope size (26 vs 22)	4.294	1.484	2.89	**0.004**	[1.36, 7.22]	0.23
*Model 4: Total energy*						
Prostate volume	0.167	13.436	2.682	**0.004**	[0.28, 0.84]	0.21
Enucleation time	1.984	0.271	7.311	** < 0.001**	[1.44, 2.52]	0.51
Resectoscope size (26 vs 22)	−1.198	8.839	−0.135	0.892	[−18.66, 16.27]	−0.01

*SE* Standard Error, *CI* Confidence Interval, *Ref* Reference

## Discussion

Beginning with Nesbit’s initiative to decrease instrument size in transurethral prostatectomy, subsequent innovations have aimed to optimize surgical feasibility and improve patient outcomes. The need for performing the transurethral surgeries with 26 Ch instruments comes from the fact that, for an efficient resection an 8 mm loop was necessary. However, to introduce a 550–1000 micron laser fiber, such huge instrument is not mandatory. Besides, during transurethral resection procedures, the irrigation fluid circulates through the bladder, therefore a huge amount of irrigation is necessary. On contrary, during AEEP the irrigation fluid only circulates in a limited space between the adenoma and the capsule. Therefore, the amount of irrigation needed is limited. With this philosophy, Figueiredo and Teloken introduced miniaturized laser AEEP equipment designed to improve the compatibility of the instruments with urethral anatomy [9]. In our series, while no urethrotomy/meatotomy was required in the MiLEP group, it was necessary in 12.5% of the patients in the standard AEEP group.

Among the most important determinants of enucleation and morcellation times are prostate volume and the experience of the surgeon [11]. When enucleation time is compared between MiLEP and HoLEP, in a study published by Schmidt et al., no significant differences in enucleation time were observed between the HoLEP and MiLEP groups [12]. In our study, however, both enucleation and operation times were shorter in the MiLEP group compared to the standard AEEP group. In Model 1, multivariable linear regression analysis demonstrated that although the strongest contributor to longer operating time was the larger prostate volume observed in the standard group, resectoscope size was also significantly associated with operating time. The longer operating time in the standard AEEP group may be attributed, at least in part, to the need for concomitant intraoperative urethrotomy performed in 10 patients. In addition, enucleation efficiency was higher in the standard AEEP group than in the MiLEP group, and resectoscope size was the only factor independently associated with this difference. However, the higher efficiency observed in the standard group may be attributable to the lower total energy settings applied in the MiLEP group (1.5 J vs. 2.0 J).

In laser AEEP procedures, the use of normal saline as an irrigation fluid prevents the development of hyponatremia or TUR syndrome, which may occur after TURP. Although a study investigating fluid absorption during HoLEP reported no changes in serum electrolytes, it demonstrated that the volume of irrigation fluid used is an independent risk factor for absorption [13]. In the comparative study of MiLEP and HoLEP by Taha et al., a significant difference in irrigation fluid consumption was observed [14]. Consistent with these findings, our study showed that irrigation fluid usage was significantly lower in the MiLEP group compared to the standard AEEP group. However, this reduction did not result in any electrolyte disturbances in patients.

Several factors, including patient age, surgeon experience, laser-related parameters, the implementation of early apical release, prostate volume, and enucleation technique, have been investigated in relation to postoperative urinary incontinence following AEEP. Furthermore, to enhance surgical effectiveness and reduce postoperative complications, numerous innovations have been introduced into the field by both surgeons and manufacturers. Another possible benefit of using smaller-caliber instruments in AEEP surgery is the potential to minimize mechanical sphincteric damage through miniaturization, thereby reducing early stress incontinence rates. Taha et al. reported that one-month incontinence rates were significantly lower in the MiLEP group. Notably, the 40% stress incontinence rate observed in the HoLEP group was higher than what has generally been reported in the literature [14]. Alves et al. conducted a study without a control group, precluding a direct comparison, while Schmidt et al. did not specifically address these factors [12, 15]. Owing to this gap in the literature, we prospectively compared both techniques in the present study. Although both incontinence and urethral stricture rates tended to be lower in the MiLEP group compared with the standard group, these differences did not reach statistical significance. A plausible explanation for this finding is that all procedures in both groups were performed by a single high-volume, experienced surgeon, which may have mitigated variability in outcomes.

These findings have important clinical implications, particularly for patients with narrow urethras or those at increased risk of sphincteric injury, in whom miniaturized instruments may offer distinct advantages. Thus, MiLEP may be considered a valuable alternative to standard AEEP. Additionally, AEEP is a size independent procedure and it has an already well defined role for especially the huge glands. On the other hand, TURP is accepted as a standard procedure especially for the small sized prostates with its world-wide availability and low cost. However, the miniaturization of the instruments with its potential to create less urethral trauma also makes laser AEEP a good option for the small sized prostates.

This study has several limitations. The relatively short postoperative follow-up period may have contributed to the absence of significant differences between the groups. In addition, the non-randomized study design resulted in differences in median prostate volume between the groups and may have introduced selection bias. Differences in laser energy settings between the groups may also have complicated the comparison of enucleation efficiency expressed as g/min. Finally, the single-surgeon design may limit the generalizability of the findings, as all procedures were performed by a highly experienced surgeon; therefore, the observed outcomes may not be directly applicable to lower-volume centers or surgeons with varying levels of experience. Multicenter, randomized prospective studies are needed to address these limitations.

## Conclusions

MiLEP with pulsed Thulium:YAG laser represents an effective modality for anatomic enucleation of the prostate. It was associated with comparable intraoperative efficiency and favorable postoperative functional outcomes compared to standard resectoscope size. Our findings suggest that MiLEP may be a suitable option for selected patients, particularly those with lower urethral compliance and smaller prostate volume.

## Data Availability

Due to the sensitive nature of the data (e.g., patient information), the datasets are not publicly available but can be shared in anonymized form upon reasonable request to the corresponding author.
